# Effectiveness of Postoperative or Preoperative Radiotherapy on Prognosis in Patients with Stage II Resectable Non-Small Cell Lung Cancer: A Retrospective Study Based on the SEER Database

**DOI:** 10.3390/medicina57111202

**Published:** 2021-11-04

**Authors:** Deng Chen, Jinming Yu

**Affiliations:** School of Public Health, Fudan University, Shanghai 200433, China; 19111020021@fudan.edu.cn

**Keywords:** postoperative radiotherapy, preoperative radiotherapy, prognosis, non-small cell lung cancer, stage II

## Abstract

*Background and Objectives*: The research on the therapeutic effect of preoperative radiotherapy (PRRT) for patients with early non-small cell lung cancer (NSCLC) is still insufficient, and the impact of postoperative radiotherapy (PORT) on the prognosis of patients with early NSCLC remains controversial. We conducted this study to investigate the effect of PORT and PRRT on prognosis for these patients. *Materials and Methods*: In total, 3640 patients with stage II NSCLC who underwent a lobectomy or pneumonectomy were extracted from the Surveillance, Epidemiology, and End Results (SEER) database. Multivariate regression was adopted to identify the independent influence of PORT or PRRT on patients’ prognosis. Subgroup analysis of survival was performed in patients with different combinations of key clinical features. We also used Kaplan-Meier analysis and competitive risk analysis to explore to which extent PORT or PRRT impacted the overall survival and cumulative mortality. *Results*: PORT was an independent risk factor of NSCLC-specific death among patients with N0 stage (HR, 1.648; 95% CI, 1.309–2.075, *p* < 0.001) and in N1 stage with <3 positive lymph nodes (HR, 2.698; 95% CI, 1.910–3.812, *p* < 0.001) in multivariate analysis. Findings from subgroup analysis for the risk of NSCLC-specific death, competitive risk analysis of NSCLC-specific cumulative mortality, and overall survival analysis also demonstrated PORT was detrimental to patients in these two subgroups above (*p* < 0.05). However, in patients with N1 stage with ≥3 positive lymph nodes, PORT may help prolong median survival. PRRT was an independent risk factor for NSCLC-specific death in multivariate analysis of patients with N0 stage (HR, 1.790; 95% CI, 1.201–2.668, *p* = 0.004), and significantly decreased overall survival in these patients (*p* < 0.001). *Conclusion*: PORT is associated with worse survival outcome and better cumulative mortality of stage II patients of NSCLC with N0 disease or N1 disease (<3 nodes), while PRRT is associated with reduced prognosis in patients with N0 stage. On the other hand, PORT may help to improve the prognosis of patients with N1 stage who have three or more lymph node metastases. Hence, PORT and PRRT should not be recommended for patients with N0 stage. However, in patients with “high volume” N1 stage, PORT might improve oncological outcomes.

## 1. Introduction

Lung cancer has the highest mortality and the second highest incidence among all cancers in the United States (US), which contributes to about 25% of all cancer deaths [1,2]. More than 85% of those cases are currently classified as non-small cell lung cancer (NSCLC), for which the predicted five-year overall survival rate is 15.9%—a figure that has only marginally improved during the past few decades [3]. It is worth mentioning that the five-year overall survival rate for NSCLC varies with tumor stage, from 60% in patients with stage IIA disease to 0–10% in patients with stage IVA-IVB disease [4]. Hence, it is of great consequence to adopt reasonable intervention or management for patients with stage I or II tumors to prevent the disease from further deterioration to the middle and late stage.

Although surgical intervention remains the gold standard for the approximately 30% of NSCLC patients who present with resectable stage II disease and who are functionally operable [5,6,7], multidisciplinary sequential therapy, which requires a delicate interplay between surgery, radiotherapy (RT), and chemotherapy (CT), turned to be the trend of management modalities [8]. Many retrospective studies have shown that postoperative RT (PORT) reduces local relapse and improves survival [9,10,11,12,13,14], and preoperative radiation (PRRT) or a combination of preoperative CT and RT is feasible with acceptable toxicity and encourages tumor downstaging and five-year survival rate in patients with clinical stage III NSCLC [15,16,17]. However, the research on the therapeutic effect of PRRT for patients with early NSCLC is lacking, and the effect of PORT on the prognosis of patients with early NSCLC remains controversial.

From February 1982 to October 1995, 296 patients with NSCLC and stage II or III disease were randomized into PORT (134 patients) and surgery alone (162 patients). PORT was administrated three–four weeks after radical operation. Irradiated fields covered the bronchial stump, ipsilateral hilum, and most of the mediastinum. Clinical data were comparable in both arms, except for the numbers of N2 patients. Results showed that the three-year and five-year overall survival rates and disease-free survival rates in the PORT group were higher than that in the surgery group, but the difference was not statistically significant (*p* = 0.56 and 0.28). A trend toward improved survival in the PORT group was observed in the patients with T3–4 N1M0 tumors, who demonstrated 20% improvement in overall survival (*p* = 0.092) and greater than 20% better disease-free survival (*p* = 0.057). These suggested that PORT significantly reduced local relapses but did not improve overall survival due to the patients’ high frequency of distant metastases [18].

B-E. Lally et al. [19] selected patients with stage II or III NSCLC from the SEER database, and only those who underwent a lobectomy or pneumonectomy and were coded as receiving PORT or observation were included. A total of 7465 patients with a median follow-up time of 3.5 years were eventually identified, according to some exclusion criteria. In this population-based cohort, they found that the use of PORT did not have a significant impact on survival. However, in subset analysis, PORT use has been associated with an increase in survival in patients with N2 nodal disease but not in patients with N1 and N0 nodal disease.

In an updated systematic review and individual patient data meta-analysis on the effectiveness of PORT for patients with NSCLC, results showed that PORT causes an 18% relative increase in the risk of death [20]. Furthermore, similar detriments were observed for local recurrence-free survival, distant recurrence-free survival, and overall recurrence-free survival. Another quantitative meta-analysis using updated information from individual participants from all randomized trials also indicated that PORT is detrimental to those with completely resected NSCLC and should not be used in the routine treatment of such patients [21].

For NSCLC patients who present with resectable stage II disease, the survival outcome of using PORT or PRRT seems to be related to some critical factors, like the use of comprehensive treatment, the N stage of the tumor, lobectomy of the lung, and number of positive lymph nodes. For this reason, we evaluated the effectiveness of PORT or PRRT by analyzing in-depth the cases with a detailed record of clinical features in the SEER database in order to provide more current information about the clinical practice of treatment for patients with NSCLC stage II disease.

## 2. Methods

**Figure 1:**
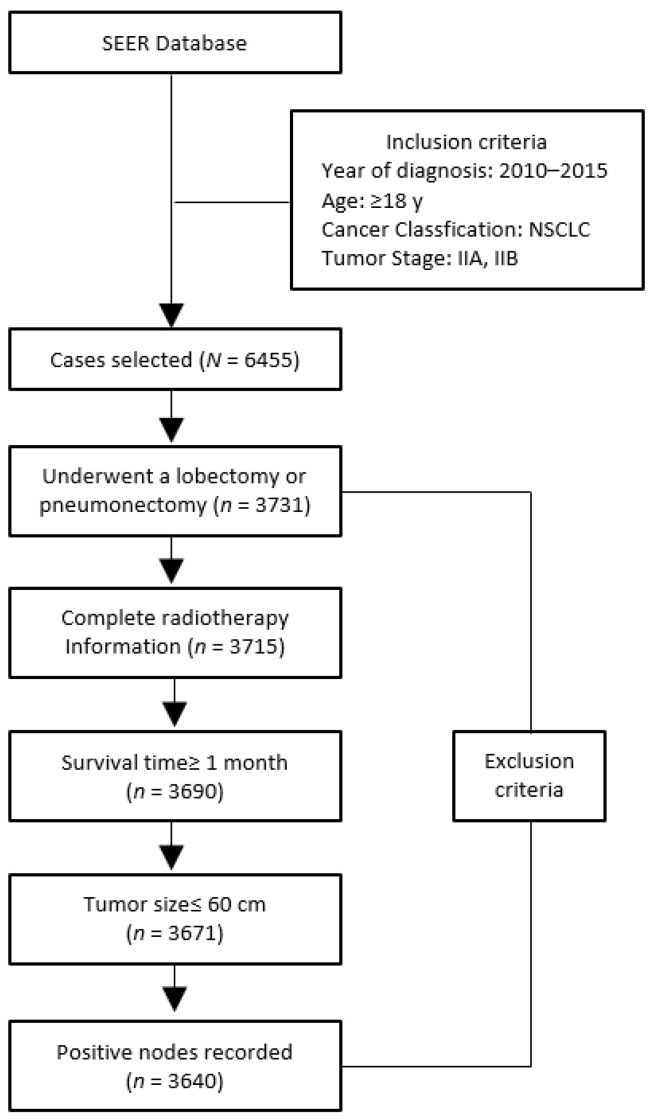
Flowchart of case inclusion and exclusion. NSCLC = non-small cell lung cancer.

The Surveillance, Epidemiology, and End Results (SEER) database, an authoritative source for cancer statistics in the US, is supported by the Surveillance Research Program (SRP) in NCI’s Division of Cancer Control and Population Sciences (DCCPS), providing information on cancer statistics in an effort to reduce the cancer burden of the nation. Since 1973, it has been accumulating data on cancer cases from various locations and sources throughout the US. The specific histologic type selected included various subtypes of large-cell carcinoma, squamous cell carcinoma, adenocarcinoma, and bronchioalveolar adenocarcinoma which were coded as 8012/3, 8013/3, 8022/3, 8031/3, 8032/3, 8033/3, 8035/3, 8046/3, 8050/3, 8052/3, 8070/3, 8071/3, 8072/3, 8073/3, 8074/3, 8082/3, 8083/3, 8084/3, 8123/3, 8140/3, 8200/3, 8201/3, 8250/3, 8251/3, 8252/3, 8253/3, 8254/3, 8255/3, 8260/3, 8310/3, 8323/3, 8333/3, 8430/3, 8480/3, 8481/3, 8490/3, 8550/3, 8560/3, 8570/3, 8574/3, and 8980/3. We identified 6455 cases according to the following admission criteria: (1) year of diagnosis from 2010 to 2015; (2) adults aged 18 years and older; (3) pathologically confirmed NSCLC; (4) tumor stage IIA or IIB (the AJCC Cancer Staging Seventh Edition). We then excluded patients who did not undergo surgery of lobectomy or pneumonectomy and had no complete radiotherapy information. In addition, given the possibility of short-term immediate death caused by the operation, patients with survival time less than 1 month were not included in this study. Furthermore, only those patients with tumor size of 60 cm or smaller were included, and we required a detailed record of the number of positive lymph nodes. Patients without other complete registration information related to our research were also excluded. Figure 1 demonstrates the flowchart of case inclusion and exclusion in detail. Eventually, we selected a total of 3640 cases as our overall population for the study.

Variables involved in our study included the basic demographic information (age at diagnosis, sex, and race), tumor-related information (year of diagnosis, pathologic grade (I–IV or unknown)), tumor size, location (upper lobe, middle lobe, lower lobe, main bronchus, or others), histology (squamous cell carcinoma, adenocarcinoma, or others), stage (IIA or IIB), T stage (T1, T2, or T3), N stage (N0 or N1), number of positive lymph nodes, treatment information (surgery, RT, and CT), and survival information (survival time, vital status, cause-specific death, and other cause of death). Age at diagnosis, tumor size, and survival month were investigated as continuous variables, while the number of positive lymph nodes was transformed into a categoric variable (<3 nodes vs. ≥3).

Key characteristics were compared between multiple groups with the use of Chi-square test, Fisher’s exact test, and analysis of variance. Prognosis comparison of different treatments was performed with Kaplan-Meier curves, and *p* value was determined using the method of log-rank (without intersection between the survival curves) or Tarone-Ware (with at least one intersection between the survival curves), respectively. Considering that the Cox regression model containing multiple variables did not satisfy the assumption of proportional hazards, we adopted a competitive risk model to explore the independent influence of different factors on survival outcome. Meanwhile, we performed competitive risk analysis to evaluate accumulative mortality of patients who received various treatments, to obtain a more accurate picture of the risk of lung cancer-specific mortality in each group. SAS 9.4 were used for data analysis, while “cmprsk”, “survival”, and “forestplot” packages in R 3.6.2 statistical software were adopted for plotting. A two-sided test was used, and a *p* value of less than 0.05 was considered as the significance level.

## 3. Results

### 3.1. Distribution Characteristic of Factors Related to Use of PORT or PRRT

A total of 3640 cases meeting the study requirements were enrolled in the study, among whom 349 received PORT and 101 received PRRT. The mean and median age at diagnosis of the patients was 67.8 (sd, 9.5) and 68.0 (range, 30–92) years. Patients with less than three positive nodes accounted for 86.5%, and the rest had three or more positive nodes.

The use of PORT/PRRT was significantly correlated with factors including age at diagnosis, pathologic grade, tumor size, tumor location, histology, stage, T stage, N stage, number of positive lymph node, and postoperative chemotherapy (POCT) use (*p* < 0.05), while it was unaffected by year of diagnosis and race (*p* > 0.05) (Table 1).

### 3.2. Univariate and Multivariate Analysis of Variables Influencing Prognosis of Patients

Tables S1 and S2 show the results of univariate regression and multivariate regression, respectively. Before or after multi-factor correction, PORT was an independent risk factor of NSCLC-specific death in the overall patients and those with N0 or N1 stage (*p* < 0.05), while PRRT was an independent risk factor merely among the overall patients and those with N1 stage (*p* < 0.05).

### 3.3. Multivariate Analysis of Important Variables Affecting Prognosis of Patients with N1 Stage

We divided patients with N1 stage into two groups by the number of positive lymph nodes, and Table 2 illustrates the results of multivariate analysis of competitive risk in each group. Given the other events or cancers that may affect the NSCLC-specific death, the independent impact factors of prognosis of patients with less three positive lymph nodes included PORT use, year of diagnosis, sex, race, pathologic grade, tumor location, histology, T stage, and postoperative chemotherapy (POCT) use, among which HR of use of PORT and PRRT (vs. neither use of PORT nor PRRT) was 2.698 (95% CI, 1.910–3.812, *p* < 0.001) and 1.453 (95% CI, 0.641–3.294, *p* = 0.370), respectively. While in patients with three or more positive lymph nodes, independent influencing factors merely contained race, tumor size, tumor location, and POCT use (all *p* < 0.05), and use of PORT (HR, 0.903; 95% CI, 0.537–1.517, *p* = 0.700) or PRRT (HR, 0.659; 95% CI, 0.076–5.639, *p* = 0.700) had no impact on patients’ NSCLC-specific death.

### 3.4. Subgroup Analysis of the Risk of Lung Cancer-Specific Death Caused by Using PORT

**Figure 2:**
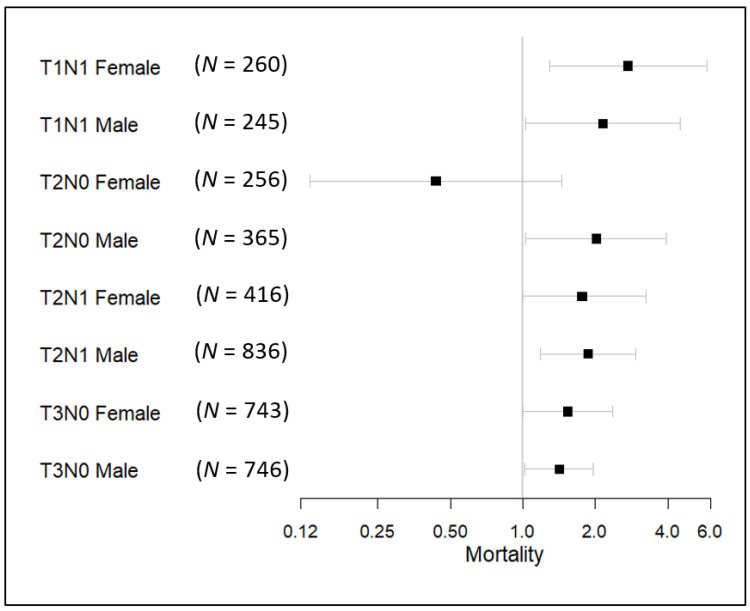
Subgroup analysis of the risk of NSCLC-specific death caused by using PORT. Note: the adjusted factors include year of diagnosis, age of diagnosis, race, pathologic grade, tumor size, position, histology, stage, and POCT use.

We performed a subgroup analysis of the risk of lung cancer-specific death in correlation with PORT. We adjusted all the other factors involved in our study that may influence the results, including the year of diagnosis, age of diagnosis, race, pathologic grade, tumor size, position, histology, stage, and POCT use. Figure 2 shows that except for the female group with T2N0, use of PORT tended to result in a worse prognosis compared with not using PORT or PRRT in the patients with various combinations of T stage, N stage, and sex (*p* < 0.05).

### 3.5. OS Analysis of Patients with N0 Stage

**Figure 3:**
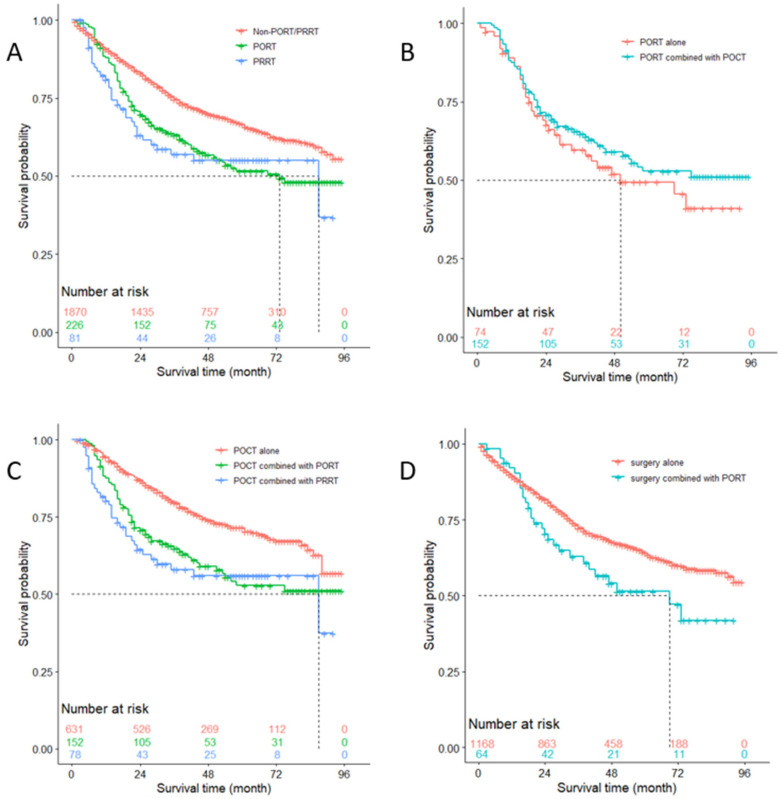
Overall survival of patients with N0 stage (**A**) who received Non-PORT/PRRT versus PORT (*p* < 0.001), Non-PORT/ PRRT versus PRRT (*p* = 0.002), and PORT versus PRRT (*p* = 0.605); (**B**) who received PORT alone versus PORT combined with POCT (*p* = 0.330); (**C**) who received POCT alone versus POCT combined with PORT (*p* < 0.001), POCT alone versus POCT combined with PRRT (*p* < 0.001), and POCT combined with PORT versus POCT combined with PRRT (*p* = 0.522); and (**D**) who featured < 3 positive lymph nodes, surgery alone versus surgery combined with PORT (*p* = 0.028). POCT = postoperative chemotherapy; PORT = postoperative radiotherapy; PRRT = preoperative radiotherapy; Non-PORT/PRRT = neither PORT nor PRRT.

Of the 2177 patients with N0 nodal disease, 1870 did not receive either PORT or PRRT, 226 received PORT, and the other 81 received PRRT. Survival curves of these patients are shown with Kaplan-Meier plots in Figure 3A. The difference of overall survival between the three groups was statistically significant; among them patients who received neither PORT nor PRRT had better survival outcome than those who received PORT (*p* < 0.001) or PRRT (*p* = 0.002), while the difference between the latter two groups was not significant (*p* = 0.605). However, median survival of patients who received PORT was 73 months, less than that of patients who received PRRT (87 months) and who received neither PORT nor PRRT (>96 months). Although no significant difference was observed between the survival curves of patients who received PORT alone and who received PORT combined with POCT (*p* = 0.330), the median survival of the former (50 months) was much less than that of the latter (>96 months) (Figure 3B).

A total of 861 patients who received POCT were divided into three groups based on whether they received PORT or PRRT. Figure 3C shows roughly that using POCT alone was associated with a better survival outcome. Log-rank test indicated that the survival difference between using POCT alone vs. using POCT combined with PORT or PRRT was statistically significant (*p* < 0.001). However, the difference of prognosis between using POCT combined with PORT and POCT combined with PRRT had no statistical significance (*p* = 0.522), albeit a different median survival (87 vs. >90 months, respectively).

We also divided patients with disease of N0 stage featuring < 3 positive lymph nodes who underwent surgery but received no POCT into two groups, stratified by whether they received PORT. It turned out that the difference between these two survival curves was statistically significant (*p* = 0.028), and the median survival of patients who received surgery combined with PORT was 69 months, less than that of patients who received surgery alone (>96 months) (Figure 3D).

### 3.6. OS Analysis of Patients with N1 Stage

**Figure 4:**
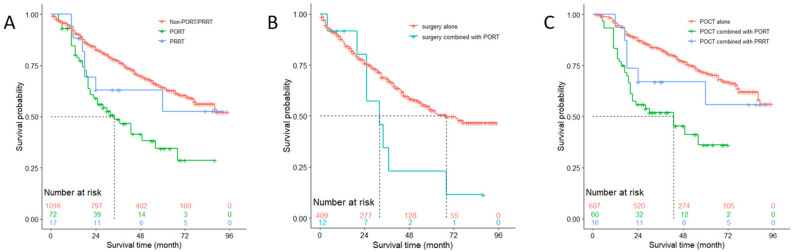
Overall survival of patients with N1 stage featured with <3 positive lymph nodes (**A**) who received Non-PORT/PRRT versus PORT (*p* < 0.001), Non-PORT/ PRRT versus PRRT (*p* = 0.471), and PORT versus PRRT (*p* = 0.204); (**B**) who received surgery alone versus surgery combined with PORT (*p* = 0.015); (**C**) who received POCT alone versus POCT combined with PORT (*p* < 0.001), POCT alone versus POCT combined with PRRT (*p* = 0.183), and POCT combined with PORT versus POCT combined with PRRT (*p* = 0.380). POCT = postoperative chemotherapy; PORT = postoperative radiotherapy; PRRT = preoperative radiotherapy; Non-PORT/PRRT = neither PORT nor PRRT.

In a similar way, the overall survival of patients with N1 stage was analyzed. Figure 4A shows that 1016 patients featuring < 3 positive lymph nodes who received neither PORT nor PRRT (median survival, >96 months) had better prognosis than those who received PORT (median survival, 34 months) (*p* < 0.001), while the survival difference between those who received neither PORT nor PRRT and who received PRRT (median survival, 34 months), and between those who received PORT and who received PRRT was of no statistical difference (both *p* > 0.05). In patients with disease of N1 stage featuring < 3 positive lymph nodes without receiving POCT, using PORT was unfavorable to prognosis compared with not using it (median survival, 32 vs. 68 months; *p* = 0.032), which is shown in Figure 4B. Among 683 patients with N1 stage who had less than three positive lymph nodes and received POCT, PORT shortened median survival compared with those who received POCT only (43 vs. >96 months, respectively; *p* < 0.001), despite the survival difference between using PRRT (median survival, >96 months) and using POCT only (*p* = 0.183), and between using PRRT and using PORT (*p* = 0.380) indicating no statistical meaning (Figure 4C).

**Figure 5:**
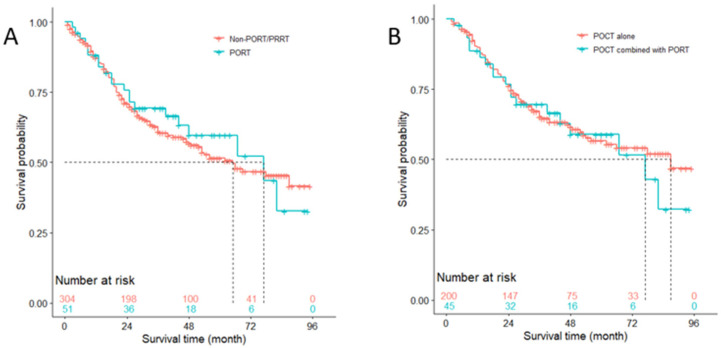
Overall survival of patients with N1 stage featured with ≥3 positive lymph nodes (**A**) who received Non-PORT/PRRT versus PORT (*p* = 0.610); (**B**) who received POCT alone versus POCT combined with PORT (*p* = 0.919). Note: POCT = postoperative chemotherapy; PORT = postoperative radiotherapy; PRRT = preoperative radiotherapy; Non-PORT/PRRT = neither PORT nor PRRT.

In 355 patients with N1 stage featuring three or more positive lymph nodes, even though using PORT did not show a significant positive impact on OS (*p* = 0.610), it prolonged the median survival from 65 months to 77 months compared with to not using PORT or PRRT (Figure 5A). Among 245 patients with N1 stage who had no less than three positive lymph nodes and who received POCT, using PORT shortened the median survival from 87 months to 77 months, though the difference between the two survival curves was not statistically significant (*p* = 0.919) (Figure 5B).

### 3.7. Competitive Risk Analysis of NSCLC-Specific Death

**Figure 6:**
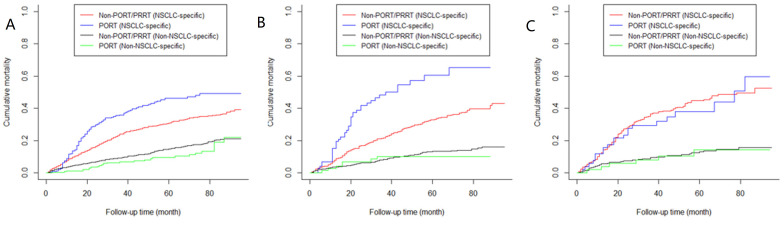
Competitive risk analysis for NSCLC-specific death of patients with (**A**) N0 stage caused by using PORT (*p* < 0.001); (**B**) N1 stage featured < 3 positive nodes caused by using PORT (*p* < 0.001); (**C**) N1 stage featured ≥ 3 positive nodes caused by using PORT (*p* = 0.783). Note: NSCLC = non-small cell lung cancer; PORT = postoperative radiotherapy; PRRT = preoperative radiotherapy; Non-PORT/PRRT = neither PORT nor PRRT.

We performed a competitive risk analysis to explore the NSCLC-specific mortality rate of patients treated with PORT and neither PORT nor PRRT. Among patients with N1 stage featuring less than three positive lymph nodes or with N0 stage, using PORT increased the NSCLC-specific deaths. Among patients who received PORT, the 1-, 3- and 5-year cumulative mortality increased by 33.33%, 50.42%, and 49.27% in patients with N0 stage (*p* < 0.001), and by 98.44%, 120.43% and 82.84% in patients with N1 stage featuring less than three positive lymph nodes (*p* < 0.001) (Figure 6A,B), respectively. There was no statistical difference between two curves of cumulative mortality of patients with N1 stage who had no less than three positive nodes (*p* = 0.0.783) (Figure 6C), however, using PORT decreased the 1-, 3- and 5-year cumulative NSCLC-specific mortality rate by 5.92%, 19.69% and 15.48%.

## 4. Discussion

Based on clinical assessment alone, patients with stage II NSCLC were considered to comprise only 5% of all patients with NSCLC [22]. The aging of the population over the years, improvement of screening technology, and promotion of health education have probably brought about the increase of incidence and discovery of stage II non-small cell lung cancer. Since lobectomy has remained the standard of care for resection of early-stage NSCLC, we selected patients who received pneumonectomy or lobectomy to be the research targets. We tried to gain insight into the effect of various treatment modalities for patients with stage II NSCLC who had different clinical features, which has been an area with controversy which needs to be solved.

A systematic review and meta-analysis of individual patient data from nine randomized controlled trials revealed a significant absolute detriment of 7% increased mortality at two years and reduced overall survival from 55% to 48% with the addition of RT after resection of stage I–III NSCLC [23]. Some key consensus and criteria do not recommend adjuvant RT for completely resected early-stage disease due to its significant adverse effect [6,24]. In our study, we also found that PORT was an independent risk factor of NSCLC-specific death among patients with N0 stage (HR, 1.648; 95% CI, 1.309–2.075, *p* < 0.001) or with N1 stage featuring less than three nodes (HR, 2.698; 95% CI, 1.910–3.812, *p* < 0.001) after adjusting other clinical characteristics. Meanwhile, the findings from subgroup analysis for the risk of lung cancer-specific death, and competitive risk analysis of lung cancer-specific cumulative mortality and overall survival analysis for patients with N0 stage or with N1 stage featuring less than three nodes support previous research as well. However, we should also pay attention to the result that, among patients with N1 stage who had no less than three positive lymph nodes, using PORT failed to independently influence the NSCLC-specific survival outcome when adjusting other related factors. Moreover, for patients in this subgroup, PORT prolonged the median survival from 65 months to 77 months, and decreased the 1-, 3- and 5-year cumulative NSCLC-specific mortality rate by 5.92%, 19.69% and 15.48% compared with not using PORT or PRRT, despite the lack of statistical significance. By contrast, however, the results from two systematic reviews illustrate that PORT is detrimental to survival in patients with stage II NSCLC [25,26]. Therefore, the number of positive lymph nodes should be considered as one of the reliable indicators of prognosis in patients with lung cancer, however, the intervention needs to be flexible according to metastases of lymph node for the patients with N1 stage.

In this study, we determined that among patients with N0 stage or with N1 stage featuring less than three nodes, PRRT compared with PORT prolonged the median survival but the difference of survival curves was of no statistical significance, and similar results occurred in the subgroups of patients with N1 stage who had <3 nodes and received POCT. This seems to indicate that PRRT may have the advantage over PORT for the improvement of patients with early stage NSCLC, even though we have no enough samples to confirm it and lack the direct support of previous studies. Nevertheless, a recent propensity matching analysis suggested that preoperative radiation may improve the outcomes of resectable IIIA/N2 NSCLC patients [17], and other studies have demonstrated that a combination of preoperative CT and irradiation benefited the tumor downstaging and survival improvement [15,16]. Furthermore, the results of another retrospective study indicated that PORT had no impact on operative mortality or morbidity [26].

Besides, our observation suggests that compared with POCT alone, POCT combined with PORT is more unfavorable to the prognosis of patients, especially for patients with N0 stage or with N1 stage featuring less than three nodes. However, when comparing with PORT alone, POCT combined with PORT betters the survival outcome (median survival, 50 vs. >96 months, respectively) of patients with N0 stage, although the difference of survival curves has no statistical significance. From the two sides above, it seems that we can determine that for early resectable NSCLC patients with or without a few lymph node metastases, the treatment should give priority to POCT alone, then POCT combined with PORT, and finally PORT alone. Results of the comparison of POCT alone and POCT combined with PRRT differ in patients with N0 stage and patients featuring less than three positive lymph nodes with N1 stage (*p* values of difference of survival curves, <0.001 vs. 0.380, respectively). We believe that the main reason may lie in that the sample size of patients with N1 stage featuring less than three nodes who received POCT combined with PRRT is small, which led to masking the difference. After all, from the overall trend of the survival curves, it turned out that POCT combined with PRRT performed worse than POCT alone.

We acknowledge the limitations of this study. First, it is a retrospective study, which unavoidably has selection bias and recall bias, although we have tried our best with statistical methodology to minimize their negative effect. Then, the sample size of some subgroups is relatively small, which may hinder us from knowing the real results of comparison between groups. We hope to enlarge the sample size in further study to answer the questions that this research cannot.

## 5. Conclusions

Our findings demonstrate that PORT correlated with lower survival and increased cumulative mortality of stage II patients of NSCLC with N0 or N1 disease (with less than 3 positive nodes) in a large population-based study. PRRT is associated with worse prognosis in the N0 stage. PORT may help to improve the prognosis of patients with N1 stage who have no less than three nodes, but more experimental studies are needed to confirm this. Further prospective clinical trials are required to prove the effect of PORT/PRRT on the survival of NSCLC patients with stage II, N1 disease.

## Figures and Tables

**Table 1:** Distribution characteristic of factors related to use of PORT or PRRT.

Variable	Non-PORT/PRRT (*n* = 3190)	PORT (*n* = 349)	PRRT (*n* = 101)	Overall (N = 3640)	*p* Value
Year of diagnosis, *n* (%)					
2010	534 (16.7)	70 (20.1)	25 (24.8)	629 (17.3)	0.263
2011	579 (18.2)	60 (17.2)	18 (17.8)	657 (18.0)	
2012	520 (16.3)	54 (15.5)	22 (21.8)	596 (16.4)	
2013	552 (17.3)	55 (15.8)	14 (13.9)	621 (17.1)	
2014	513 (16.1)	61 (17.5)	11 (10.9)	585 (16.1)	
2015	492 (15.4)	49 (14.0)	11 (10.9)	552 (15.2)	
Age at diagnosis					
Mean (SD)	67.9 (9.4)	66.9 (9.7)	64.8 (9.3)	67.8 (9.5)	<0.001
Median (Min, Max)	68.0 (30.0, 92.0)	67.0 (35.0, 88.0)	66.0 (36.0, 85.0)	68.0 (30.0, 92.0)	
Sex, *n* (%)					
Female	1521 (47.7)	149 (42.7)	39 (38.6)	1709 (47.0)	0.049
Male	1669 (52.3)	200 (57.3)	62 (61.4)	1931 (53.0)	
Race, *n* (%)					
Black	290 (9.1)	32 (9.2)	15 (14.9)	337 (9.3)	0.052
White	2654 (83.2)	278 (79.7)	80 (79.2)	3012 (82.7)	
Others	246 (7.7)	39 (11.2)	6 (5.9)	291 (8.0)	
Pathologic Grade, *n* (%)					
Grade I	369 (11.6)	16 (4.6)	4 (4.0)	389 (10.7)	<0.001
Grade II	1343 (42.1)	137 (39.3)	22 (21.8)	1502 (41.3)	
Grade III	1221 (38.3)	168 (48.1)	46 (45.5)	1435 (39.4)	
Grade IV	69 (2.2)	7 (2.0)	2 (2.0)	78 (2.1)	
Unknown	188 (5.9)	21 (6.0)	27 (26.7)	236 (6.5)	
Tumor size					
Mean (SD)	45.3 (33.1)	46.1 (26.8)	57.2 (22.5)	45.7 (32.4)	0.001
Median (Min, Max)	40.0 (1.00, 540)	40.0 (1.00, 225)	54.0 (15.0, 140)	40.0 (1.00, 540)	
Location, *n* (%)					
Upper lobe	1769 (55.5)	226 (64.8)	80 (79.2)	2075 (57.0)	<0.001
Middle lobe	157 (4.9)	14 (4.0)	0 (0)	171 (4.7)	
Lower lobe	1146 (35.9)	92 (26.4)	16 (15.8)	1254 (34.5)	
Main bronchus	26 (0.8)	6 (1.7)	1 (1.0)	33 (0.9)	
Multi-lobe	92 (2.9)	11 (3.2)	4 (4.0)	107 (2.9)	
Histology, *n* (%)					
Ad	2064 (64.7)	180 (51.6)	43 (42.6)	2287 (62.8)	<0.001
Sq	958 (30.0)	143 (41.0)	47 (46.5)	1148 (31.5)	
Others	168 (5.3)	26 (7.4)	11 (10.9)	205 (5.6)	
Stage, *n* (%)					
IIA	1706 (53.5)	129 (37.0)	25 (24.8)	1860 (51.1)	<0.001
IIB	1484 (46.5)	220 (63.0)	76 (75.2)	1780 (48.9)	
T, *n* (%)					
T1	460 (14.4)	43 (12.3)	3 (3.0)	506 (13.9)	<0.001
T2	1451 (45.5)	109 (31.2)	27 (26.7)	1587 (43.6)	
T3	1279 (40.1)	197 (56.4)	71 (70.3)	1547 (42.5)	
N, *n* (%)					
N0	1870 (58.6)	226 (64.8)	81 (80.2)	2177 (59.8)	<0.001
N1	1320 (41.4)	123 (35.2)	20 (19.8)	1463 (40.2)	
Positive Lymph Nodes, n (%)					
<3	2787 (87.4)	273 (78.2)	89 (88.1)	3149 (86.5)	<0.001
≥3	403 (12.6)	76 (21.8)	12 (11.9)	491 (13.5)	
POCT, *n* (%)					
No	1752 (54.9)	92 (26.4)	5 (5.0)	1849 (50.8)	<0.001
Yes	1438 (45.1)	257 (73.6)	96 (95.0)	1791 (49.2)	

Note: POCT = postoperative chemotherapy; PORT = postoperative radiotherapy; PRRT= preoperative radiotherapy; Non-PORT/PRRT = neither PORT nor PRRT. In classification of histology, Ad = adenocarcinoma, Sq = squamous cell carcinoma.

**Table 2:** Multivariate analysis of important variables affecting prognosis of patients in N1 stage.

	<3 Positive Lymph Nodes (*n* = 1105)			≥3 Positive Lymph Nodes (*n* = 358)		
Variables	HR	95 CI	*p* Value	HR	95 CI	*p* Value
Treatment						
Non-PORT/ PRRT		Ref			Ref	
PORT	2.698	(1.910, 3.812)	<0.001	0.903	(0.537, 1.517)	0.700
PRRT	1.453	(0.641, 3.294)	0.370	0.659	(0.076, 5.693)	0.700
Year of diagnosis						
2010		Ref			Ref	
2011	0.889	(0.651, 1.213)	0.460	0.955	(0.585, 1.559)	0.850
2012	0.951	(0.687, 1.315)	.760	1.088	(0.633, 1.871)	0.760
2013	0.663	(0.470, 0.937)	0.020	0.934	(0.517, 1.690)	0.820
2014	0.707	(0.487, 1.026)	0.068	0.757	(0.420, 1.362)	0.350
2015	0.532	(0.338, 0.837)	0.006	0.716	(0.364, 1.410)	0.330
Age at diagnosis	1.009	(0.997, 1.020)	0.150	1.015	(0.996, 1.034)	0.130
Sex						
Female		Ref			Ref	
Male	1.328	(1.073, 1.644)	0.009	1.137	(0.786, 1.647)	0.500
Race						
Black		Ref			Ref	
White	0.683	(0.499, 0.933)	0.017	0.387	(0.197, 0.762)	0.006
Others	0.588	(0.377, 0.916)	0.019	1.145	(0.595, 2.201)	0.690
Pathologic Grade, n ()						
I		Ref			Ref	
II	1.302	(0.801, 2.117)	0.290	0.809	(0.362, 1.808)	0.610
III	1.748	(1.075, 2.843)	0.024	1.122	(0.522, 2.412)	0.770
IV	1.779	(0.737, 4.297)	0.200	1.414	(0.386, 5.176)	0.600
Unknown	0.904	(0.401, 2.036)	0.810	0.7	(0.246, 1.994)	0.500
Tumor Size	1.001	(0.998, 1.003)	0.670	1.019	(1.001, 1.038)	0.035
Location						
Upper lobe		Ref			Ref	
Middle lobe	0.952	(0.645, 1.404)	0.800	1.031	(0.515, 2.065)	0.930
Lower lobe	0.986	(0.780, 1.246)	0.900	1.419	(1.001, 2.011)	0.049
Main bronchus	0.819	(0.278, 2.411)	0.720	0.721	(0.125, 4.168)	0.710
Multi-lobe	2.587	(1.318, 5.075)	0.006	1.428	(0.624, 3.266)	0.400
Histology						
Ad		Ref			Ref	
Sq	0.763	(0.597, 0.975)	0.030	0.689	(0.458, 1.036)	0.073
Others	0.858	(0.520, 1.415)	0.550	1.023	(0.505, 2.072)	0.950
Stage						
IIA		Ref			Ref	
IIB	0.963	(0.689, 1.346)	0.820	0.642	(0.344, 1.200)	0.160
T						
T1		Ref			Ref	
T2	1.334	(1.051, 1.694)	0.018	0.999	(0.631, 1.583)	1.000
POCT						
No		Ref			Ref	
Yes	0.640	(0.510, 0.803)	<.001	0.664	(0.456, 0.967)	0.033

Note: POCT = postoperative chemotherapy; PORT = postoperative radiotherapy; PRRT = preoperative radiotherapy; Non-PORT/PRRT = neither PORT nor PRRT. In classification of histology, Ad = adenocarcinoma, Sq = squamous cell carcinoma.

## Data Availability

The data and materials that support the findings of this study are available from SEER database, an authoritative source for cancer statistics in the US, which is supported by the Surveillance Research Program (SRP) in NCI’s Division of Cancer Control and Population Sciences (DCCPS).

## Supplementary Materials

The following are available online at https://www.mdpi.com/article/10.3390/medicina57111202/s1, Table S1: Univariate analysis of variables influencing prognosis of patients, Table S2: Multivariate analysis of variables influencing prognosis of patients.
