# Supplementary material for: Effectiveness of Postoperative or Preoperative Radiotherapy on Prognosis in Patients with Stage II Resectable Non-Small Cell Lung Cancer: A Retrospective Study Based on the SEER Database
Source: Medicina (Kaunas). 2021 Nov 4;57(11):1202. doi: 10.3390/medicina57111202 (PMC8624228; doi:10.3390/medicina57111202)
Supplement: Supplementary file 1 [file medicina-57-01202-s001.zip › medicina-1399525-supplementary.pdf]

Table S1 Univariate analysis of variables influencing prognosis of patients

	N0			N1			Overall		
Variables	HR	95% CI	*P* Value	HR	95% CI	*P* Value	HR	95% CI	*P* Value
Total, n		2177			1463			3640	
Treatment									
Non-PRRT/PORT		Ref			Ref			Ref	
PORT	1.600	(1.290, 1.980)	<.001	1.720	(1.322, 2.250)	<.001	1.620	(1.370, 1.920)	<.001
PRRT	1.620	(1.120, 2.340)	.010	1.180	(0.566, 2.440)	.660	1.480	(1.060, 2.050)	.020
Year of diagnosis									
2010		Ref			Ref			Ref	
2011	0.837	(0.665, 1.052)	.130	0.973	(0.763, 1.242)	.830	0.894	(0.756, 1.057)	.190
2012	0.914	(0.724, 1.154)	.450	0.968	(0.739, 1.268)	.810	0.930	(0.78, 1.109)	.420
2013	1.019	(0.809, 1.283)	.870	0.725	(0.544, 0.967)	.029	0.892	(0.746, 1.067)	.210
2014	0.658	(0.501, 0.865)	.003	0.703	(0.517, 0.954)	.024	0.675	(0.551, 0.828)	<.001
2015	0.575	(0.417, 0.793)	.001	0.566	(0.394, 0.815)	.002	0.571	(0.449, 0.726)	<.001
Age at diagnosis	1.020	(1.010, 1.020)	<.001	1.010	(1.005, 1.024)	.072	1.015	(1.009, 1.021)	<.001
Sex									
Female		Ref			Ref			Ref	
Male	1.370	(1.180, 1.590)	<.001	1.240	(1.040, 1.470)	.016	1.311	(1.171, 1.468)	<.001
Race									
Black		Ref			Ref			Ref	
White	0.969	(0.754, 1.240)	.800	0.827	(0.633, 1.079)	.160	0.897	(0.730, 1.221)	.240
Others	1.299	(0.924, 1.830)	.130	0.632	(0.426, 0.937)	.022	0.944	(0.748, 1.077)	.660
Pathologic Grade									
I		Ref			Ref			Ref	
II	1.140	(0.899, 1.450)	.280	1.190	(0.805, 1.805)	.390	1.180	(0.963, 1.446)	.110
III	1.430	(1.124, 1.810)	.004	1.630	(1.075, 2.416)	.016	1.535	(1.254, 1.878)	<.001

IV	2.020	(1.245, 3.270)	.004	1.820	(0.862, 3.448)	.094	1.965	(1.325, 2.915)	<.001
Unknown	1.430	(1.022, 1.990)	.037	1.000	(0.503, 1.689)	.990	1.303	(0.972, 1.747)	.076
Tumor Size	1.004	(1.002, 1.005)	<.001	1.007	(1.003, 1.014)	.003	1.003	(1.002, 1.004)	<.001
Location									
Upper lobe		Ref			Ref			Ref	
Middle lobe	1.140	(0.753, 1.720)	.540	0.991	(0.710, 1.380)	.960	1.090	(0.842, 1.411)	.510
Lower lobe	1.260	(1.076, 1.470)	.004	1.093	(0.907, 1.320)	.350	1.191	(1.056, 1.343)	.004
Main bronchus	2.170	(1.055, 4.480)	.035	0.818	(0.338, 1.980)	.660	1.372	(0.788, 2.389)	.260
Others	1.770	(1.203, 2.590)	.004	1.897	(1.137, 3.160)	.014	1.786	(1.314, 2.428)	<.001
Histology									
Ad		Ref			Ref				Ref
Sq	1.180	(1.010, 1.390)	.041	1.010	(0.678, 1.500)	.960	1.055	(0.932, 0.195)	.390
Others	1.620	(1.190, 2.190)	.002	0.897	(0.738, 1.090)	.270	1.340	(1.053, 1.705)	.017
Stage									
IIA		Ref			Ref			Ref	
IIB	1.110	(0.938, 1.307)	.230	1.230	(0.969, 1.560)	.089	1.028	(0.919, 1.150)	.630
T									
T1		Ref			Ref			Ref	
T2	—	—	—	1.340	(1.120, 1.610)	.002	1.197	(1.006, 1.423)	.042
T3	1.107	(0.938, 1.307)	.230	—	—	—	1.104	(0.926, 1.315)	.270
POCT									
No		Ref			Ref			Ref	
Yes	0.932	(0.800, 1.090)	.370	0.699	(0.587, 0.832)	<.001	0.861	(0.769, 0.963)	.009
Positive Lymph Nodes									
<3		Ref			Ref			Ref	
⩾3	1.670	(1.270, 2.210)	<.001	1.420	(1.170, 1.710)	<.001	1.520	(1.309, 1.765)	<.001

Note: POCT = postoperative chemotherapy; PORT = postoperative radiotherapy; PRRT= preoperative radiotherapy; Non-PORT/PRRT = neither PORT nor PRRT. In classfication of histology, Ad = adenocarcinoma, Sq = squamous cell carcinoma.

Table S2 Multivariate analysis of variables influencing prognosis of patients

	N0			N1			Overall		
Variables	HR	95% CI	*P* Value	HR	95% CI	*P* Value	HR	95% CI	*P* Value
Total, n		2177			1463			3640	
Treatment									
Non-PRRT/PORT		Ref			Ref			Ref	
PORT	1.648	(1.309, 2.075)	<.001	1.721	(1.276, 2.320)	<.001	1.662	(1.388, 1.989)	<.001
PRRT	1.790	(1.201, 2.668)	.004	1.340	(0.630, 2.851)	.450	1.694	(1.197, 2.398)	.003
Year of diagnosis									
2010		Ref			Ref			Ref	
2011	0.875	(0.691, 1.107)	.270	0.967	(0.749, 1.249)	.800	0.908	(0.764, 1.078)	.270
2012	0.884	(0.693, 1.129)	.320	1.031	(0.781, 1.360)	.830	0.940	(0.785, 1.126)	.500
2013	1.045	(0.824, 1.326)	.720	0.742	(0.553, 0.997)	.048	0.908	(0.756, 1.091)	.300
2014	0.684	(0.517, 0.905)	.008	0.734	(0.539, 1.000)	.050	0.692	(0.563, 0.850)	<.001
2015	0.633	(0.457, 0.877)	.006	0.600	(0.413, 0.872)	.007	0.611	(0.478, 0.780)	<.001
Age at diagnosis	1.017	(1.008, 1.026)	<.001	1.009	(0.999, 1.019)	.072	1.014	(1.008, 1.021)	<.001
Sex									
Female		Ref			Ref			Ref	
Male	1.278	(1.089, 1.500)	.003	1.258	(1.052, 1.504)	.012	1.263	(1.122, 1.421)	<.001
Race									
Black		Ref			Ref			Ref	
White	0.951	(0.737, 1.226)	.700	0.758	(0.574, 1.002)	.052	0.855	(0.710, 1.029)	.098
Others	1.247	(0.877, 1.773)	.220	0.534	(0.356, 0.802)	.003	0.844	(0.646, 1.102)	.210
Pathologic Grade									
I		Ref			Ref			Ref	
II	1.187	(0.927, 1.520)	.170	1.205	(0.805, 1.805)	.370	1.236	(1.004, 1.522)	.046
III	1.423	(1.099, 1.843)	.007	1.612	(1.075, 2.416)	.021	1.561	(1.262, 1.930)	<.001

IV	2.017	(1.192, 3.412)	.009	1.724	(0.862, 3.448)	.120	2.067	(1.375, 3.108)	<.001
Unknown	1.249	(0.878, 1.778)	.220	0.921	(0.503, 1.689)	.790	1.201	(0.889, 1.622)	.230
Tumor Size	1.003	(1.002, 1.004)	<.001	1.007	(0.996, 1.018)	.230	1.003	(1.002, 1.004)	<.001
Location									
Upper lobe		Ref			Ref			Ref	
Middle lobe	1.184	(0.778, 1.807)	.430	1.061	(0.759, 1.482)	.730	1.163	(0.895, 1.510)	.260
Lower lobe	1.287	(1.090, 1.519)	.003	1.106	(0.916, 1.337)	.300	1.213	(1.071, 1.373)	.002
Main bronchus	1.740	(0.764, 3.966)	.190	0.897	(0.351, 2.296)	.820	1.347	(0.743, 2.442)	.330
Others	1.699	(1.145, 2.520)	.009	1.828	(1.077, 3.101)	.025	1.700	(1.241, 2.328)	.001
Histology									
Ad		Ref			Ref				Ref
Sq	0.959	(0.800, 1.149)	.650	0.732	(0.593, 0.903)	.004	0.856	(0.748, 0.979)	.024
Others	1.226	(0.878, 1.711)	.230	0.870	(0.579, 1.308)	.500	1.063	(0.825, 1.369)	.640
Stage									
IIA		Ref			Ref			Ref	
IIB	1.134	(0.953, 1.350)	.160	0.856	(0.591, 1.240)	.410	1.079	(0.844, 1.380)	.540
T									
T1		—			Ref			Ref	
T2	—	—	—	1.179	(0.921, 1.508)	.190	1.028	(0.856, 1.234)	.770
T3	—	—	—	—	—	—	0.904	(0.661, 1.236)	.530
POCT									
No		Ref			Ref			Ref	
Yes	0.847	(0.713, 1.005)	.057	0.663	(0.548, 0.802)	<.001	0.799	(0.705, 0.907)	<.001
Positive Lymph Nodes									
<3		Ref			Ref			Ref	
⩾3	1.674	(1.243, 2.255)	<.001	1.368	(1.115, 1.679)	.003	1.538	(1.310, 1.806)	<.001

Note: POCT = postoperative chemotherapy; PORT = postoperative radiotherapy; PRRT= preoperative radiotherapy; Non-PORT/PRRT = neither PORT nor PRRT. In classfication of histology, Ad = adenocarcinoma, Sq = squamous cell carcinoma.
